# Increased circulating FGF21 level predicts the burden of metabolic demands and risk of vascular diseases in adults with type 2 diabetes

**DOI:** 10.1186/s12902-023-01523-y

**Published:** 2023-12-07

**Authors:** Zhen Liu, Yue Peng, Supeng Li, Yusheng Lin, Yunfeng Huang, Wenting Chen, Chunhua Bao, Zengxian Zhou, Zhuofeng Lin, Liangmiao Chen

**Affiliations:** 1 School of Pharmaceutical College, Wenzhou Medical University, Wenzhou, China; 2 Department of Cardiology, The 1st affiliated Hospital of Wenzhou Medical Unversity, Wenzhou, China; 3 Department of Endocrinology, The 3rd affiliated Hospital of Wenzhou Medical University (Ruian People’s Hospital, Wenzhou, China; 4 Laboratory Animal Center of Wenzhou Medical University, Wenzhou, China

**Keywords:** Fibroblast growth factor 21, Vascular Diseases, Carotid Atherosclerosis, Hypertension, Type 2 Diabetes Mellitus

## Abstract

**Objectives:**

Type 2 diabetes mellitus (T2DM) is a chronic metabolic disorder characterized by chronic hyperglycemia and metabolic stress, involved in the stepwise development of cardiovascular complications. Fibroblast growth factor 21 (FGF21) is a novel hepatokine involved in regulating glucose and lipid metabolism, and has been linked to the prediction, treatment, and improvement of prognosis in multiple cardiovascular diseases (CVDs). The aim of this study is to explore the relationship between FGF21 levels and vascular diseases (VDs) including carotid atherosclerosis (CAS) and hypertension (HP) in patients with T2DM.

**Methods:**

Baseline serum FGF21 was determined in a cross-sectional study of 701 patients with T2DM and 258 healthy control.

**Results:**

The morbidity of CAS was increased in T2DM patients with HP as compared with those without (*p* < 0.001). The average serum FGF21 level of healthy was [123.9 (67.2-219.3)]. Baseline FGF21 was significantly higher in those who developed CAS or HP than in those who did not [305.9 (177.2-508.4) vs. 197.2 (129.7-308.3) pg/mL, *p* < 0.001]. In addition, an elevated serum FGF21 was observed in T2DM patients with HP and CAS than that of T2DM patients with CAS or HP [550.5 (312.6-711.3) vs. 305.9 pg/mL, *p* < 0.001]. Serum FGF21 levels were positively correlated with body mass index and carotid intima media thicknes (*p* < 0.05), the association remained significant after adjusting for age and T2DM duration. Furthermore, the multinomial logistic regression showed that serum FGF21 was independently associated with CAS and HP in patients with T2DM after adjustment for demographic and traditional VDs risk factors (*p* < 0.001).

**Conclusions:**

Baseline FGF21 is elevated in VDs during diabetes, changes of serum FGF21 levels were appropriately matched to metabolic stress. FGF21can be used as an independent predictor for diagnosing VDs and predicting prognosis.

**Supplementary Information:**

The online version contains supplementary material available at 10.1186/s12902-023-01523-y.

## Introduction

Diabetes is one of the most prevalent and serious metabolic diseases, characterized by chronic hyperglycemia. Hyperglycemia induces various stress conditions including metabolic stress, oxidative stress, and endoplasmic reticulum stress, promotes pathological changes accompanied by impaired cellular function and tissue damage, leading to inflammatory disorders in vascular system and the development of cardiovascular diseases (CVDs), that cannot be reversed by simple control of blood glucose levels [1, 2]. CVDs remains a major cause of mortality and morbidity of patients with type 2 diabetes mellitus (T2DM). Compared with the general population, patients with T2DM had earlier onset, more severe and more extensive lesions of CVDs [3]. Therefore, the prevention and treatment of vascular diseases (VDs) is of great significance for patients with T2DM.

Fibroblast growth factor 21 (FGF21) is an atypical member of FGF family which functions as an endocrine hormone in controlling metabolic multiorgan crosstalk enhancing energy expenditure through glucose and lipid metabolism, and playing an increasingly important role in the prediction, treatment, and improvement of prognosis in CVDs [4]. Human FGF21 is a polypeptide composed of 209 amino acid residues, which is mainly secreted by liver and adipose tissue, and functions in an endocrine and paracrine manner by connecting with transmembrane albumen β through carboxylated terminal. Therefore, transmembrane protein β determine the tissue specificity of FGF21. Recently, FGF21 has evolved to a major metabolic regulator in increasing insulin sensitivity and then reducing blood sugar levels, improving lipid profile and promoting fatty acid oxidation, lowering blood pressure, increasing energy consumption through various signaling pathways [5–7]. Some outstanding achievements on the research of FGF21 in hypertension (HP) and atherosclerosis (AS) have been obtained [8, 9]. Our previous studies have found that FGF21 has beneficial effects in protecting against angiotensin II (Ang II)-induced HP and vascular dysfunction by ativation of Ang II-converting enzyme 2/Ang II-(1–7) axis through fine-tuning the multi-organ crosstalk among liver, adipose tissue, kidney, and blood vessels [8]. We also found that FGF21 can prevents AS by suppression of hepatic sterol regulatory element-binding protein-2 and induction of adiponectin in mice [9]. Correspondingly, subsequent clinical studies have also confirmed that FGF21 can be used as a biomarker for HP and subclinical AS, and is of particular interest as potential therapeutic agents for the treatment of those CVDs [10–12]. These results imply that FGF21 could regulate cytokines response under various stressed conditions.

Chronic hyperglycemia in diabetes patients is often accompanied by dyslipidemia, insulin resistance, obesity, and inflammatory reaction, as well as HP, which for sure are all risk factors for AS [13]. On the one hand, blood flow directly acts on the blood vessel wall, leading to an increase in transmural pressure and the resulting damage of endothelial cells may help increasing of vascular intima permeability and the enhancement of ultrafiltration of low-density lipoprotein (LDL) and albumin, thus making it easy to enter the vascular intima. On the other hand, blood flow and pressure can also lead to the proliferation and hypertrophy of vascular smooth muscle cells in the middle layer and the increase of extracellular matrix collagen content, leading to the densification of the middle membrane, thus obstructing the transport of LDL and deposition in the intima [14]. In HP, stimulated by relevant factors, the vascular endothelial function is impaired with a reducing of synthesis and/or release of endothelial relaxing factors, and accelerating of inactivation of those factors, thus the sensitivity of vascular smooth muscle to vascular endothelial relaxing factors is reduced, while the vascular endothelial contraction factor is relatively increased, resulting in hyperplasia and hypertrophy of vascular smooth muscle cell, thickening of intima media thickness (IMT), deposition of lipids, and the decline of vascular elastic function, finally, the atherosclerotic plaques can be gradually formed [14, 15]. At the same time, AS is increasingly being recognized as a systemic vascular disease, which can cause the contraction of vascular smooth-muscle cell, and increase the resistance of peripheral vascular, thus in return leading to an increase in blood pressure [15]. Taken together, these findings suggest that diabetes, HP, and AS affect each other adversely and worsen existing pathophysiological conditions.

FGF21 is involved in the pathological changes of diabetes, AS, and HP, respectively. Abnormally elevated blood sugar levels can gradually lead to VDs including AS and HP. Diabetes, HP and AS interact and form a vicious cycle under metabolic stress conditions. However, whether FGF21 resistance are influenced by AS and HP in patients with T2DM remains known. Therefore, the aim of this study was to investigate the associations of carotid AS (CAS) and HP with FGF21 responses during stressed conditions.

## Materials and methods

### Participants

This was a cross-sectional study conducted in the Third Affiliated Hospital of Wenzhou Medical University (Ruian People’s Hospital) from July 1, 2019 to January 28, 2023. A total of 701 patients clinically diagnosed with T2DM were enrolled when hospitalized or referred to the Endocrinology Outpatient Clinic, and 258 healthy control subjects (blood donors) were prospectively included. The clinical diagnosis of T2DM referred to the standards of diabetic diagnosis and classification put forward by the World Health Organization (WHO) in 1999: symptoms of diabetes and casual plasma glucose (PG) levels ≥ 11.1 mmol/L, fasting plasma glucose (FPG) levels ≥ 7.0 mmol/L, or 2-h PG levels ≥ 11.1 mmol/L following a 75 g oral glucose tolerance test [16]. HP was defined as systolic blood pressure (SBP) ≥ 140 mmHg and/or diastolic blood pressure (DBP) ≥ 90 mmHg after monitoring blood pressure three times on different days without the use of antihypertensive medications, or the patients had a history of HP who were currently taking antihypertensive drugs even though his blood pressure was lower than 149/90 mmHg. Participants with type 1 diabetes, or gestational diabetes, with intracranial, vertebral, or subclavian artery disease, with history of carotid endarterectomy or carotid stent implantation were excluded. Detailed information of subjects including age, gender, history of tobacco and alcohol use, history of diseases, and medication use history were recorded by a standardized questionnaire. Anthropometric parameters including body weight, height, SBP, and DBP. Body mass index (BMI) was calculated as body weight (kg)/height^2^ (m^2^) thereafter.

### Measurement of biochemical index and FGF21

A collection of whole blood samples following an 8–12 h period of fasting from all participants were used to isolate serum samples and then stored in -80 °C for subsequent analysis. Fasting plasma glucose (FPG), triglyceride (TG), total cholesterol (TC), high-density lipoprotein cholesterol (HDL-c), LDL cholesterol (LDL-c), and serum creatinine levels were measured by an auto biochemical analyzer (Cobas c702; Roche, Shanghai, China). Hemoglobin A1c (HbA1c) was determined using high-performance liquid chromatography method (BioRad; Hercules, CA, USA). Serum creatinine levels, age, gender, and race were used to calculate estimated glomerular filtration rate (eGFR) with the chronic kidney disease-epidemiology collaboration (CKD-EPI) equation [17]. Circulating FGF21 levels were quantified using ELISA (Antibody and Immunoassay Service; HKU, HK). The intra- and inter- assay coefficients of variation were 4.5% and 6.9%, respectively.

### Assessment of CAS

Considering that increased carotid IMT (cIMT) is a sign of AS, which can be used as a marker reflecting systemic and cardiovascular risk factors [18], in this study, CAS monitored by color doppler ultrasound (HDI 5000, Philips Medical Systems, USA) with a 7.5 MHz probe was used to evaluate the cIMT, carotid plaque crouse score, and the number of unstable plaque of all the subjects. Measurement of IMT: Three times measurement of the patient’s bilateral common carotid artery, bifurcation of carotid artery, internal and external carotid artery was carried out along the long axis of the blood vessels, and the average thickness was taken. The numerical value using crouse score [19] to judge cIMT thickness has not been unified at present. In this project, 0.9 mm < cIMT < 1.2 mm was considered as thickening, and cIMT ≥ 1.2 mm was considered as plaque formation. Ignoring the length of each plaque, the maximum thickness of isolated plaques in the blood vessels as mentioned above was added to obtain the sum of carotid atherosclerotic plaque integrals. Endometrial thickening and plaque formation are classified as CAS.

### Statistical analysis

Statistical analyses were performed by using SPSS 26.0 software (SPSS Inc., Chicago, IL, USA). Continuous data were described with median (interquartile range), or mean ± standard deviation. Categorical data were expressed as absolute numbers and/or percentages. The continuous data among patients between more than two groups were compared with one-way ANOVA (for parametric data) or Kruskal-Wallis test (for nonparametric data) according to the normality or homogeneity of the variance test, and chi-square for categorical data. Chi-square test and Kendall tau-b correlation analysis were used to identify the association between CAS and HP. Pearson correlation coefficient and Kendall tau-b correlation coefficient were used to analyze the correlation of FGF21 with CAS and HP parameters in patients with T2DM. Partial correlation analysis adjusted for age and T2DM duration was performed to determine the association of FGF21 with clinical variables. Correlation coefficient among the variables was calculated. In addition, multinomial logistic regression was applied to examine the association between FGF21 and CAS and HP using different models. Two-sided *p*-values < 0.05 indicated statistical significance. Additionally, GraphPad Prism 8 (GraphPad Softwares Inc., La Jolla, CA, USA) was used to draw statistical graph.

## Results

### HP and CAS correlate with each other in patients with T2DM

A total of 701 T2DM patients were enrolled in this study, 323 subjects with HP (46.1%), 353 subjects with CAS (50.4%), 214 subjects with both HP and CAS (30.5%). Moreover, the prevalence of CAS in 701 patients with T2DM was 50.87%, and the prevalence of CAS in T2DM subjects with HP is significantly higher than those without HP (66.3% vs. 33.7%, *p* < 0.001) (Table 1), suggesting HP may be a risk factor to promote the morbidity of CAS in patients with T2DM. Further correlation analysis confirmed that HP positively correlated with CAS parameters including left cIMT, right IMT, and plaque score (all *p* < 0.001) (Supplementary Table 1).

**Table 1:** Relationship between CAS and HP in T2DM patients

	Non-HP	HP	*χ* ^2^	*p*
Non-CAS	239 (63.2%)	109 (33.7%)	60.56	< 0.001
CAS	139 (36.8%)	214 (66.3%)		

Note. CAS: carotid atherosclerosis; HP: hypertension

### Serum FGF21 levels are significantly increased in T2DM patients with CAS and/or HP

To investigate whether FGF21 is related to the pathogenesis of CAS in patients with T2DM, we first explored the influence factors of CAS in these T2DM subjects. As shown in Table 2, an increased manner of age and T2DM duration were observed in these T2DM subjects with CAS and/or HP compared with those without. Similarly, a worse of renal function index (eGFR) was found in these T2DM individuals with HP and/or CAS compared with those without (all *p* < 0.001). Consistent with those changes, a higher level of blood pressure including SBP and DBP was observed, and more antihypertensive drugs use in these individuals, following with a higher BMI, left IMT, right IMT value, and plaque score. However, no obvious differences of hypercholesterolemia, hypotriglyceridemia, and lipid-lowering therapy, as well as blood lipid parameters including TG, TC, HDL-c and LDL-c were observed in these T2DM subjects with or without those VDs, respectively (all *p* > 0.05). In addition, a higher hypoglycemic agents use was found in these T2DM subjects with CAS and/or HP compared with those without, as a consequence, no obvious difference of FPG, but a lower HbA1c were found for the well-controlled diabetes.

**Table 2:** Comparison of clinical characteristic in T2DM patients with or without CAS and HP compliations

Variables	Healthy (n = 258)	T2DM (n = 239)	CAS or HP (n = 248)	CAS with HP (n = 214)	*p*
FGF21, pg/mL	123.9 (67.2-219.3)	197.2 (129.7-308.3)	305.9 (177.2-508.4)	550.5 (312.6-711.3)	**< 0.001**
BMI, kg/m^2^	22.28 ± 2.92	24.982 ± 3.35	25.537 ± 3.01	26.315 ± 3.27	**< 0.001**
Male, %	41.9	72.0	75.8	76.6	**< 0.001**
Current smoking, %	16.6	43.5	32.7	35	**< 0.001**
Current drinking, %	19.5	30.5	33.5	24.8	**0.004**
Age, years	41.36 ± 12.25	46.41 ± 10.55	54.53 ± 8.77	58.39 ± 7.27	**< 0.001**
T2DM duration, months	/	52.50 ± 66.68	73.35 ± 82.76	111.05 ± 88.75	**< 0.001**
FPG, mmol/L	4.82 (4.51–5.11)	7.5 (6.4–9.5)	7.6 (6.2–9.53)	7.1 (6.0-8.9)	0.165
HbA1c, %	5.4 (5.17–5.6)	8.7 (7.25–11.1)	8.4 (7.3–10.5)	7.9 (7.1–9.6)	**0.002**
SBP, mmHg	117.84 ± 13.04	126.55 ± 16.48	134.13 ± 19.59	144.12 ± 19.96	**< 0.001**
DBP, mmHg	70.49 ± 9.09	75.22 ± 10.31	76.14 ± 11.34	77.86 ± 11.37	**< 0.001**
TG, mmol/L	1.13 (0.81–1.57)	1.5 (1.1–2.3)	1.7 (1.1–2.3)	1.7 (1.2–2.4)	0.200
TC, mmol/L	4.95 (4.32–5.51)	4.7 (4.0-5.35)	4.7 (4.08–5.5)	4.6 (3.9–5.3)	0.622
HDL-c, mmol/L	1.36 (1.14–1.54)	1.1 (0.9–1.3)	1.0 (0.9–1.2)	1.0 (0.9–1.2)	0.414
LDL-c, mmol/L	3.11 (2.56–3.72)	2.9 (2.3–3.4)	2.9 (2.3–3.4)	2.7 (2.1–3.5)	0.216
ALT, U/L	18 (13–26)	56 (19–206)	38.5 (19–164)	27 (16-96.5)	**< 0.001**
AST, U/L	67 (55–80)	83.6 (66–90)	84 (63–92)	76 (60-90.08)	0.110
ALP, U/L	20 (17-23.25)	29 (17–42,7)	25 (17.25–40.28)	21 (16-39.23)	**0.010**
eGFR, ml/min/1.73m^2^	118.5 (109.2-128.4)	111 (102.8-118.5)	105 (96.6-110.7)	100.3 (89.7-106.8)	**< 0.001**
Antihypertensive therapy, %	/	6.3	23.4	63.1	**< 0.001**
Left cIMT	/	0.76 ± 0.18	1.07 ± 0.21	1.21 ± 0.78	**< 0.001**
Right cIMT	/	0.74 ± 0.20	0.96 ± 0.24	1.08 ± 0.22	**< 0.001**
Plaque score	/	0.17 ± 0.38	1.32 ± 1.10	2.33 ± 0.72	**< 0.001**
Hypercholesterolemia, %	/	10.5	8.1	10.7	0.553
Hypotriglyceridemia, %	/	25.1	25.8	31.3	0.273
Lipid-lowering therapy, %	/	25.9	25.8	32.7	0.178
No, %	/	35.1	29.4	17.8	**< 0.001**
Insulin, %	/	6.7	10.1	5.6	0.159
OHA, %	/	42.7	36.7	41.1	0.377
Insulin + OHA, %	/	15.5	25	35.5	**< 0.001**

Note. Data are presented as mean ± SD, or median (interquartile range); FGF21: fibroblast growth factor 21; BMI: body mass index; FPG: fasting plasma glucose; HbA1c: haemoglobin A1c; SBP: systolic blood pressure; DBP: diastolic blood pressure; TG: triglyceride; TC: total cholesterol; HDL-c: high-density lipoprotein cholesterol; LDL-c: low-density lipoprotein cholesterol; eGFR: estimated glomerular filtration rate; cIMT: carotid intima media thickness; No: no hypoglycemic agents use; OHA: oral hypoglycemic agent; CAS: carotid atherosclerosis; HP: hypertension

**Fig. 1:**
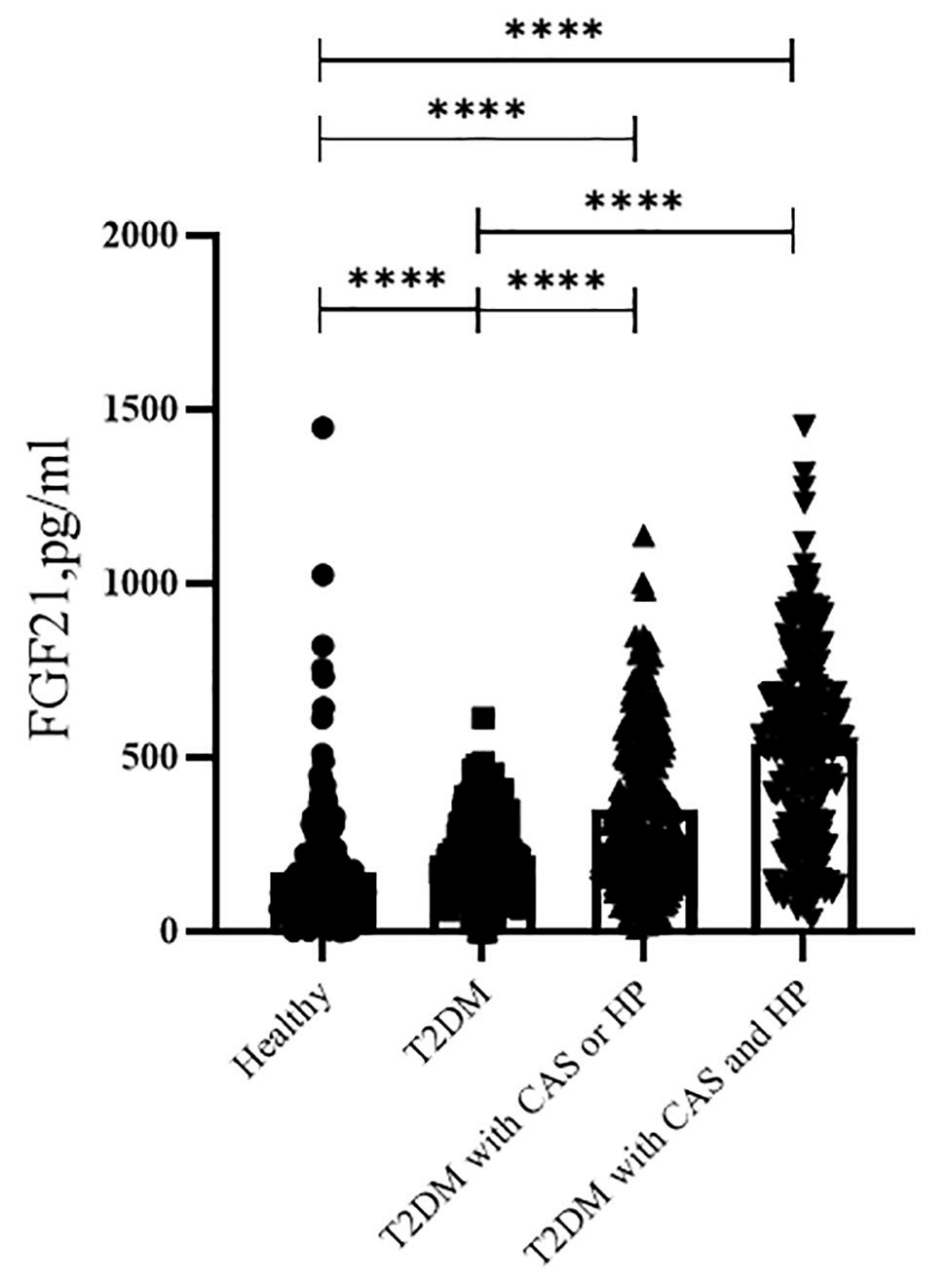
Serum FGF21 levels in T2DM patients with or without relevant VDs including CAS and/or HP

Interestingly, multiple comparisons results showed that serum FGF21 levels were significantly increased in these T2DM patients with CAS or HP as compared with those without [305.9 (177.2-508.4) vs. 123.9 (67.2-219.3) pg/mL, *p* < 0.001] (multiple comparisons’ data not shown). Futhermore, an evidently higher of serum FGF21 levels was also observed in these T2DM patients with both CAS and HP as compared to those with one of these VDs [550.5 (312.6-711.3) vs. 305.9 (177.2-508.4) pg/mL, *p* < 0.001] (multiple comparisons’ data not shown) (Table 2; Fig. 1), suggesting that elevated serum FGF21 levels during diabetes may be related to the progress of VDs.

### Serum FGF21 levels are closely correlated with a cluster of CAS parameters and HP in patients with T2DM

We next investigated the correlations between the expression levels of serum FGF21 and various conventional clinical risk indexes and CAS parameters adjusted by age and T2DM duration. Pearson’s correlation indicated that there is no significant correlation between FGF21 levels and parameters such as FPG, HbA1c, and serum lipid parameters including TC, HDL-c, LDL-c,ALT and ALP. However, FGF21 in the serum has highly significant positive correlation with BMI, age, TG ,antihypertensive agents use, plaque score,left cIMT, and right cIMT, respectively (all *p* < 0.05), but not with gender, SBP, DBP, or eGFR after adjustment for age (all *p* > 0.05). Finally, it is worth mentioning that the positive correlation of serum FGF21 with BMI, TG, left cIMT, right cIMT, antihypertensive agents use and plaque score still remained significant even after the adjustment for age and duration of T2DM (Table 3).

**Table 3:** Partial correlation of serum FGF21 with other risk factors of CAS and HP

Variables	Serum FGF21^*^			Serum FGF21^*^ (age-adjusted)			Serum FGF21^*^ (age-and T2DM duration-adjusted)	
	*r*	*p*		*r*	*p*		*r*	*p*
BMI	0.212	**< 0.001**		0.279	**< 0.001**		0.279	**< 0.001**
Male	0.108	**0.004**		0.059	0.304		0.059	0.304
Age	0.197	**< 0.001**		/	/		/	**/**
T2DM duration	0.072	0.058		-0.005	0.936		/	**/**
FPG^*^	0.006	0.884		-0.013	0.818		-0.014	0.812
HbA1c^*^	-0.058	0.124		-0.012	0.756		-0.012	0.757
SBP	0.196	**< 0.001**		0.085	0.406		0.085	0.411
DBP	0.098	**0.01**		0.108	0.294		0.110	0.285
TG^*^	0.155	**< 0.001**		0.192	**0.001**		0.192	**0.001**
TC^*^	-0.021	0.572		-0.015	0.792		-0.015	0.791
HDL-c^*^	-0.069	0.070		-0.109	0.056		-0.109	0.056
LDL-c^*^	-0.072	0.057		-0.064	0.262		-0.065	0.259
eGFR^*^	-0.193	**< 0.001**		-0.095	0.097		-0.096	0.094
ALT^*^	-0.039	0.666		0.051	0.376		0.060	0.295
ALP^*^	0.042	0.266		0.028	0.625		0.028	0.623
Antihypertensive therapy	0.330	**< 0.001**		0.315	**< 0.001**		0.331	**< 0.001**
Left cIMT	0.226	**< 0.001**		0.212	**< 0.001**		0.212	**< 0.001**
Right cIMT	0.338	**< 0.001**		0.355	**< 0.001**		0.355	**< 0.001**
Plaque score	0.308	**< 0.001**		0.426	**< 0.001**		0.430	**< 0.001**

Note. ^*^ Log transformed before analysis. FGF21: fibroblast growth factor 21; BMI: body mass index; FPG: fasting plasma glucose; HbA1c: haemoglobin A1c; SBP: systolic blood pressure; DBP: diastolic blood pressure; TG: triglyceride; TC: total cholesterol; HDL-c: high-density lipoprotein cholesterol; LDL-c: low-density lipoprotein cholesterol; eGFR: estimated glomerular filtration rate; cIMT: carotid intima media thickness; ALT: alanine transaminase; AST: aspartate Transaminase; ALP: alkaline phosphatase; CAS: carotid atherosclerosis; HP: hypertension

### Serum FGF21 levels are independently associated with CAS and HP in patients with T2DM

The multinomial logistic regression analysis was performed in this study, to further explore whether serum FGF21 was the independent predictor of the risk of CAS and HP in patients with T2DM. We noticeably found that serum FGF21 was independently associated with CAS or HP [*OR* 2.066 (*95%CI* 1.543–2.766), *p* < 0.001], together with age, gender, SBP, and smoking in model 1 adjustment for basic factors. Furthermore, serum FGF21 and antihypertensive agents use were found to be independently associated with CAS or HP in model 2 adjustment for T2DM complications and drug use. In addition, serum FGF21 was independently associated with CAS or HP, together with SBP, DBP, and eGFR in model 3 adjusted by biochemical variables. Finally, a full model was developed adjustment for risk factors with *p* < 0.05 in models 1–3 and all the variables significantly correlated with serum FGF21, to adequately investigate whether serum FGF21 was the independent risk factor for CAS and/or HP in patients with T2DM. Likewise, the significant association remained between serum FGF21 and CAS or HP in full model (Table 4).

**Table 4:** Multinomial logistic regression showing whether FGF21 was an independent influencing factor for CAS and HP

Variables	T2DM vs. CAS or HP			T2DM vs. CAS with HP	
	*OR* (95%CI)	*p*		*OR* (95%CI)	*p*
**Model 1**					
FGF21^*^	2.066 (1.543–2.766)	**< 0.001**		6.311 (4.251–9.37)	**< 0.001**
Age	1.091 (1.064–1.118)	**< 0.001**		1.142 (1.105–1.181)	**< 0.001**
Gender	2.115 (1.294–3.458)	**0.003**		4.141 (2.257–7.597)	**< 0.001**
T2DM duration	1.001 (0.998–1.004)	0.422		1.005 (1.002–1.008)	**0.003**
BMI	1.058 (0.988–1.132)	0.105		1.125 (1.037–1.22)	**0.005**
SBP	1.02 (1.003–1.038)	**0.024**		1.042 (1.021–1.062)	**< 0.001**
DBP	0.984 (0.957–1.012)	0.261		0.977 (0.945–1.009)	0.163
Smoking	1.642 (1.069–2.522)	**0.024**		1.42 (0.84–2.4)	0.191
Drinking	0.678 (0.434–1.06)	0.088		0.983 (0.56–1.725)	0.952
**Model 2**					
FGF21^*^	1.855 (1.428–2.41)	**< 0.001**		4.763 (3.281–6.915)	**< 0.001**
Antihypertensive therapy	0.29 (0.156–0.54)	**< 0.001**		0.071 (0.038–0.133)	**< 0.001**
Antidiabetic therapy					
No	0.379 (0.062–2.303)	0.292		1.625 (0.104–25.353)	0.729
Insulin	0.242 (0.036–1.637)	0.146		1.806 (0.102–31.877)	0.687
OHA	0.395 (0.065–2.406)	0.314		1.221 (0.078–19.049)	0.887
Insulin + OHA	0.186 (0.029–1.187)	0.075		0.437 (0.027–7.038)	0.559
Hypercholesterolemia	1.466 (0.659–3.262)	0.349		1.046 (0.413–2.65)	0.924
Hypotriglyceridemia	1.088 (0.5-2.368)	0.831		1.317 (0.52–3.335)	0.561
Lipid-lowering therapy	0.913 (0.384–2.168)	0.836		0.727 (0.261–2.027)	0.542
**Model 3**					
FGF21^*^	5.068 (2.573–9.985)	**< 0.001**		4.984 (2.530–9.815)	**< 0.001**
FPG^*^	1.115 (0.251–4.967)	0.886		0.120 (0.016–0.899)	**0.039**
HbA1c^*^	0.401 (0.048–3.333)	0.398		0.054 (0.002–1.304)	0.072
SBP	1.030 (1.014–1.047)	**< 0.001**		1.063 (1.039–1.087)	**< 0.001**
DBP	0.966 (0.941–0.992)	**0.010**		0.966 (0.932–1.002)	0.063
TG^*^	0.779 (0.196–3.093)	0.723		1.015 (0.151–6.839)	0.988
TC^*^	0.816 (0.002-308.053)	0.948		1.377(0.001-1664.835)	0.636
HDL-c^*^	0.250 (0.017–3.643)	0.392		0.063 (0.002–2.202)	0.128
LDL-c^*^	1.126 (0.033–38.203)	0.843		0.587 (0.009–38.282)	0.803
ALT^*^	0.284 (0.109–0.738)	0.010		0.262 (0.070–0.980)	**0.047**
AST^*^	9.050 (1.100-74.469)	0.041		7.813 (0.450-135.588)	0.158
ALP^*^	1.526 (0.223–10.457)	0.667		0.785 (0.002–1.304)	0.859
eGFR^*^	0.094 (0.021–0.427)	**0.002**		0.021 (0.004–0.107)	**< 0.001**
**Full model**					
FGF21^*^	4.600 (2.225–9.510)	**< 0.001**		4.601 (2.225–9.510)	**< 0.001**
Age	1.104 (1.073–1.135)	**< 0.001**		1.191 (1.129–1.257)	**< 0.001**
Gender	2.119 (1.276–3.519)	**0.004**		4.907 (2.115–11.387)	**< 0.001**
T2DM duration	1.000 (0.996–1.003)	0.873		1.001 (0.995–1.007)	0.798
BMI	1.041 (0.968–1.121)	0.278		1.111 (0.986–1.251)	0.083
SBP	1.011 (0.998–1.023)	**0.095**		1.034 (1.014–1.055)	**0.001**
FPG	1.438 (0.344–6.019)	0.619		0.530 (0.052–5.379)	0.591
Smoking	1.301 (0.842–2.011)	0.236		1.151 (0.562–2.358)	0.701
Antihypertensive therapy	0.302 (0.147–0.623)	**0.001**		2.679(0.436–16.457)	**0.001**
ALT^*^	0.713 (0.427–1.192)	0.197		0.806 (0.317–2.054)	0.652
eGFR^*^	12.801(0.259-632.546)	0.200		19.983(0.091-4398.562)	0.277

Note. ^*^ Log transformed before analysis

Model 1: adjusted by basic factors including age, male, T2DM duration, BMI, SBP, DBP, smoking, and drinking; Model 2: adjusted by complications and drug use; Model 3: adjusted by biochemical variables; Full Model, adjusted by variables with *p* < 0.05 in models 1–3

Simillarly, data from multinomial logistic regression analysis between T2DM group and CAS plus HP group also demonstrated that serum FGF21 was independently associated with CAS and HP in four different models (Table 4). Futhermore, pairwise comparison among groups indicated that serum FGF21 level was significantly elevated in these T2DM patients with those two kind of VDs as compared with those without CAS (*p* < 0.001). However, there was no significant difference for FGF21 between T2DM group and T2DM with CAS group (*p* = 0.089), as well as T2DM with HP group compared to T2DM with HP and CAS group (*p* = 0.198) (Supplementary Tables 2–3). The morbidity of CAS appears likely to be given the occurrence of HP in patients with T2DM. To determine whether FGF21 is related to CAS independent of HP, partial correlation of serum FGF21 with CAS adjusted by HP was performed. CAS related parameters including CAS, left cIMT, and right cIMT were all in correlation with FGF21 levels when adjusted by HP in the correlation analysis (all *p* < 0.001) (Supplementary Table 4). Taken together, those data indicated that serum FGF21 is independently associated with HP and CAS in patients with T2DM, moreover, CAS is related to FGF21 independent of HP.

## Discussion

In the current study, we demonstrated that T2DM patients with VDs including CAS and HP have higher FGF21 level than those without. There was a strongly positive significant correlation of serum FGF21 with BMI, left cIMT, and right cIMT in the total diabetes sample. This correlation was preserved even after adjustment for age and duration of diabetes. This agrees with previous study reported that higher serum FGF21 levels were significantly elevated, and associated with diabetic complications including carotid artery plaque [20]. AS usually found after the occurrence of cardiovascular events such as HP, myocardial infarction, and stroke, which causes long-term hidden damage to important organs. HP has been recognized as an important factor promoting the formation and progression of AS. The incidence of CAS in patients with HP is more than three times elevated as those without, both SBP and DBP are all closely related to CAS. Similar results were found in our study, all the CAS parameters incluging left cIMT, right cIMT, and plaque score are closely connected with HP. The rise of SBP for long-term is considered to be the main risk factor for the increase of cIMT. Accordingly, management of elevated SBP can reduce the degree of CAS and stenosis progression [21]. Therefore, the early detection of pathophysiological changes prior to clinical symptoms is of great value for the prevention of atherosclerotic CVDs. High levels of circulating FGF21 may be a compensatory reaction to offset VDs.

In recent years, the effects of FGF21 on anti-inflammation, reducing tissue damage, and regulation of glucose and lipid metabolism have become a focus of research interest. Our previous basic research found that FGF21 can exert anti-inflammatory and anti-injury effects through adiponectin dependent and independent pathways [22]. The organism activates PPAR-α factor pathway to increase FGF21 level [23]. Serum FGF21 was elevated in patients with carotid artery plaque than those without [20]. Moreover, in our previous study we have shown that serum FGF21 is increased in patients with coronary heart disease and independently associated with adverse lipid profile [24]. In present study, we evaluated the changes of serum FGF21 level in patients with T2DM and diabetes-associated vascular complications including CAS and HP, and healty control. Evidence showed that serum FGF21 levels were significantly elevated in patients with T2DM than healthy control, and much higher levels of FGF21 were observed in T2DM patients combined with CAS and/or HP, similar results to other studies [10, 25, 26]. Logistic regression analysis showed that serum FGF21 levels in T2DM population was independently associated with CAS and HP. After adjusting for HP, FGF21 levels were still significantly correlated with cIMT levels, which indicated that FGF21 is related to CAS independent of HP in the population of T2DM. In addition, the blood pressure indicators including SBP and DBP, and CAS parameters including cIMT and plaque score were significantly increased in these T2DM individuals with CAS and/or HP compared with those without those VDs. Those data suggested that FGF21 increases in VDs during diabetes possibly response to accelerated metabolic demands.

The mechanism of elevated FGF21 under various stress conditions including diabetes, CAS, and HP still under study. The possible mechanism for this action due to the fact that FGF21 via binding to its specific receptors 1 (FGFR1) before physiologically regulate global metabolism, and this process requires the synergistic action of transmembrane protein β [27]. However, under the injury induced by these pathological lesions, the expression of transmembrane protein β was inhibited [28], as a result, FGF21 increased compensatively for anti-injury and anti-inflammation.

There is increasing evidence that FGF21 can be used as a biomarker and new therapeutic tool for HP and subclinical AS. In present study, serum levels of FGF21 in T2DM patients with HP was significantly increased than those without HP. FGF21 is an independent risk factor for T2DM patients with CAS or HP, and is positively correlated with obesity-related index (BMI), and the degree of AS index (IMT), indicating that FGF21 is increased in the condition of obesity, CAS, HP and other cardiovascular and metabolic diseases, and is a potent regulator of glucose and lipid metabolism. Chronic treatment with recombinant FGF21 could improve lipid metabolism in obese mice via suppression of the lipogenic gene named srebp-1. In addition, FGF21 analog (LY2045319) was discovered to be a metabolic regulator which has beneficial metabolic effects in reducing serum LDL-c level and increasing HDL-c level in obese human subjects with T2DM [29, 30]. Our previous work highlights the important role of the antihypertensive treatment prepared by FGF21 involving in the improvement of HP caused by Ang II, which may provide a fundamental for clinical application of FGF21 in HP [12]. However, studies have also shown that FGF21 may not have a cholesterol-lowering effect under normal lipid levels. Under the condition of dyslipidemia, a risk factor for atherosclerosis, HDL alone cannot improve the blood lipid profile of the body, and in this case, the compensatory increase of FGF21 level plays a role in improving blood lipid by increasing the reverse transport of cholesterol [31]. Simillary, there is no significant correlation between FGF21 and hyperlipidemia parameters including TG, TC, HDL-c, and LDL-c in this study. Therefore, whether FGF21 can directly improve CAS by regulating blood lipid needs further verification.

This is a cross-sectional study without follow-up of the subjects, therefore, the specific role of FGF21 in human body needs further investigation. Serum level of FGF21 was increased, following a diagnosis of VDs including CAS and HP during diabetes, which is possibly a response to accelerated metabolic demands. The exploration of the correlation and predictive value of FGF21 level in T2DM, CAS and HP may provide evidence for further selection of FGF21 analogities instead of drugs in the treatment of metabolic syndrome and its related cardiovascular and cerebrovascular diseases.

### Limitations

Nevertheless, the present study has several limitations. Our study is partly limited by the lack of measurements for coronary artery disease, in which serum FGF21 concentration is reported significantly higher [32]. Therefore, further studies are needed to elucidate the impact of different kinds of CVDs on the association of FGF21 and metabolic VDs.

### Electronic supplementary material

Below is the link to the electronic supplementary material.

Supplementary Material 1

## Data Availability

Trial data of this study are available from the corresponding authors on reasonable request. The original data may be obtained from the Lead Contact Dr. Xuemian Lu (the 3rd Affiliated Hospital of Wenzhou Medical University (Ruian People’s Hospital); Email: 13,705,871,118@163.com).
