# Supplementary material for: Increased circulating FGF21 level predicts the burden of metabolic demands and risk of vascular diseases in adults with type 2 diabetes
Source: BMC Endocr Disord. 2023 Dec 7;23:272. doi: 10.1186/s12902-023-01523-y (PMC10702049; doi:10.1186/s12902-023-01523-y)
Supplement: Supplementary file 1 — Supplementary Material 1 [file 12902_2023_1523_MOESM1_ESM.docx]

**Supplementary Data**

**Supplementary Table 1** Relationship between CAS parameters and HP

|  | HP | |
| --- | --- | --- |
| Variables | *r* | *p* |
| Left cIMT | 0.226 | < 0.001 |
| Right cIMT | 0.234 | < 0.001 |
| Plaque score | 0.283 | < 0.001 |
| CAS | 0.294 | < 0.001 |

**Supplementary Table 2** Comparison between groups with CAS and/or HP

| Variables | T2DM  (n = 239) | CAS  (n = 139) | CAS with HP  (n = 214) | HP  (n = 109) | *p* |
| --- | --- | --- | --- | --- | --- |
| FGF21, pg/mL | 123.9 (67.2-219.3) | 227.8 (140.2-354.7) | 550.5 (312.6-711.3) | 476.2 (305.3-629.1) | < 0.001 |
| Left cIMT | 0.76 ± 0.18 | 1.16 ± 0.14 | 1.21 ± 0.78 | 0.84 ± 0.18 | < 0.001 |
| Right cIMT | 0.74 ± 0.20 | 1.04 ± 0.23 | 1.08 ± 0.22 | 0.82 ± 0.20 | < 0.001 |
| Plaque score | 0.17 ± 0.38 | 2.17 ± 0.61 | 2.33 ± 0.72 | 0.24 ± 0.43 | < 0.001 |

**Supplementary Table 3** Pairwise comparison between groups of FGF21

| Pairwise comparison | *SE* | *p* |
| --- | --- | --- |
| T2DM vs. CAS | 21.601 | 0.089 |
| T2DM vs. HP | 23.405 | < 0.001 |
| T2DM vs. CAS with HP | 19.058 | < 0.001 |
| CAS vs. HP | 25.908 | < 0.001 |
| CAS vs. CAS with HP | 22.06 0 | < 0.001 |
| HP vs. CAS with HP | 23.830 | 0.198 |

**Supplementary Table 4** Partial correlation of serum FGF21 with CAS adjusted by HP

|  | Serum FGF21^*^ (HP-adjusted) | |
| --- | --- | --- |
| Variables | *r* | *p* |
| CAS | 0.257 | < 0.001 |
| Left cIMT | 0.182 | 0.001 |
| Right cIMT | 0.308 | < 0.001 |

Note. ^*^ Log transformed before analysis.
